# Sorbitol mediates age-dependent changes in apple plant growth strategy through gibberellin signaling

**DOI:** 10.1093/hr/uhae192

**Published:** 2024-07-11

**Authors:** Xumei Jia, Shuo Xu, Fei Wang, Yiwei Jia, Yubin Qing, Tengteng Gao, Zhijun Zhang, Xiaomin Liu, Chao Yang, Fengwang Ma, Chao Li

**Affiliations:** State Key Laboratory for Crop Stress Resistance and High-Efficiency Production/Shaanxi Key Laboratory of Apple, College of Horticulture, Northwest A&F University, Yangling 712100, Shaanxi, China; State Key Laboratory for Crop Stress Resistance and High-Efficiency Production/Shaanxi Key Laboratory of Apple, College of Horticulture, Northwest A&F University, Yangling 712100, Shaanxi, China; State Key Laboratory for Crop Stress Resistance and High-Efficiency Production/Shaanxi Key Laboratory of Apple, College of Horticulture, Northwest A&F University, Yangling 712100, Shaanxi, China; State Key Laboratory for Crop Stress Resistance and High-Efficiency Production/Shaanxi Key Laboratory of Apple, College of Horticulture, Northwest A&F University, Yangling 712100, Shaanxi, China; State Key Laboratory for Crop Stress Resistance and High-Efficiency Production/Shaanxi Key Laboratory of Apple, College of Horticulture, Northwest A&F University, Yangling 712100, Shaanxi, China; State Key Laboratory for Crop Stress Resistance and High-Efficiency Production/Shaanxi Key Laboratory of Apple, College of Horticulture, Northwest A&F University, Yangling 712100, Shaanxi, China; State Key Laboratory for Crop Stress Resistance and High-Efficiency Production/Shaanxi Key Laboratory of Apple, College of Horticulture, Northwest A&F University, Yangling 712100, Shaanxi, China; State Key Laboratory for Crop Stress Resistance and High-Efficiency Production/Shaanxi Key Laboratory of Apple, College of Horticulture, Northwest A&F University, Yangling 712100, Shaanxi, China; State Key Laboratory for Crop Stress Resistance and High-Efficiency Production/Shaanxi Key Laboratory of Apple, College of Horticulture, Northwest A&F University, Yangling 712100, Shaanxi, China; State Key Laboratory for Crop Stress Resistance and High-Efficiency Production/Shaanxi Key Laboratory of Apple, College of Horticulture, Northwest A&F University, Yangling 712100, Shaanxi, China; State Key Laboratory for Crop Stress Resistance and High-Efficiency Production/Shaanxi Key Laboratory of Apple, College of Horticulture, Northwest A&F University, Yangling 712100, Shaanxi, China

## Abstract

Plants experience various age-dependent changes during juvenile to adult vegetative phase. However, the regulatory mechanisms orchestrating the changes remain largely unknown in apple (*Malus domestica*). This study showed that tissue-cultured apple plants at juvenile, transition, and adult phase exhibit age-dependent changes in their plant growth, photosynthetic performance, hormone levels, and carbon distribution. Moreover, this study identified an age-dependent gene, sorbitol dehydrogenase (*MdSDH1*), a key enzyme for sorbitol catabolism, highly expressed in the juvenile phase in apple. Silencing *MdSDH1* in apple significantly decreased the plant growth and GA3 levels. However, exogenous GA3 rescued the reduced plant growth phenotype of TRV-*MdSDH1*. Biochemical analysis revealed that MdSPL1 interacts with MdWRKY24 and synergistically enhance the repression of MdSPL1 and MdWRKY24 on *MdSDH1*, thereby promoting sorbitol accumulation during vegetative phase change. Exogenous sorbitol application indicated that sorbitol promotes the transcription of *MdSPL1* and *MdWRKY24*. Notably, MdSPL1-MdWRKY24 module functions as key repressor to regulate GA-responsive gene, Gibberellic Acid-Stimulated *Arabidopsis* (*MdGASA1*) expression, thereby leading to a shift from the quick to the slow-growth strategy. These results reveal the pivotal role of sorbitol in controlling apple plant growth, thereby improving our understanding of vegetative phase change in apple.

## Introduction

Many perennial woody plants experience a long juvenile phase before flowering [1–3]. The transition from the juvenile to the adult phase, referred to as the vegetative phase change, is highly regulated by various endogenous cues such as plant age, sugars, and phytohormones [4, 5]. Vegetative phase change of plants leads to a series of morphology and physiology changes, including leaf morphology, photosynthetic traits, sink-source balances, leaf hormone dynamics, and growth strategies [5–8]. For example, changes in the leaf shape and trichome appearance of leaves are the most commonly used markers of vegetative phase change in *Arabidopsis* [7, 8]. In Myrtaceae (*Eucalyptus globulus ssp. globulus*), vegetative phase change is accompanied by an increase in the leaf cuticle thickness, stomatal density, and the size of leaf blade and the disappearance of epicuticular wax in the leaves [9]. Juvenile *Populus tremula x alba* leaves also have a higher leaf nitrogen, specific leaf area (SLA), and mean mass-based photosynthetic rates than adult leaves [10]. SLA, the ratio of leaf area to dry mass, is determined by the thickness of the leaf blade and cell density; thus, a high SLA contributes to the photosynthetic performance of the plants [10, 11]. Recent studies have shown that leaf photosynthetic changes alter plant growth and cause a switch from the fast- to the slow-growth strategy during the vegetative phases of *Arabidopsis thaliana*, *Zea mays*, and *P. tremula x alba* [12]. However, the regulatory mechanisms underlying age-related changes in the growth strategies of perennial woody plants remain unclear.

In many species, a highly conserved microRNA, miR156, in the aging pathway regulates vegetative phase change [10, 13, 14]. High miR156 levels suppress the expression of *SQUAMOSA PROMOTER BINDING PROTEIN-LIKEs* (*SPLs*) to sustain the juvenile phase [5, 8]. Evidence suggests that the miR156-SPL module is vital in diverse developmental processes [6, 14]. For example, *SPL9* and *SPL3* change the leaf size through their effect on *BLADE-ON-PETIOLE1* (*BOP1*) and *BOP2* expression [15]. *Arabidopsis SPLs* regulate trichome distribution on the leaf blade by affecting miR172 levels [16, 17]. Moreover, *TaSPL3/17* regulates wheat tillering by integrating the strigolactone signaling pathway [18].

Sugars are also key age-dependent internal signals for vegetative phase change [19, 20]. Sucrose, glucose, and fructose promote vegetative phase change by suppressing *MIR156A/C* expression [20, 21]. Arabidopsis chlorophyll-deficient mutant *chlorina1–4* (*ch1–4*) reduces the photosynthetic rate and delays vegetative phase change, but exogenous glucose can restore this phenotype [19, 21]. Moreover, plant sucrose levels regulate the timing of *Arabidopsis* vegetative phase change via the trehalose 6-phosphate (T6P) pathway [22]. *TREHALOSE PHOSPHATE SYNTHASE1* (*TPS1*) is a key enzyme in T6P synthesis. Thus, the loss of *TPS1* increases *MIR156A/C* expression and prolongs the juvenile phase in *Arabidopsis* [22, 23]. In the Rosaceae family, sorbitol is a special photosynthetic product in the leaves of many fruit trees, and it can be transformed into glucose and fructose by sorbitol dehydrogenase (SDH) [24–26]. Several studies have demonstrated that sorbitol is a key signal regulating flower bud formation in loquat (*Eriobotrya japonica*) [27], pollen tube growth [28], and resistance against *Alternaria alternata* in apple [29]. However, the role of sorbitol in regulating vegetative phase change in fruit trees remains unknown.

Some transcription factors (TFs), such as SPL, WRKY, and MYB family members, are critical in multiple developmental processes in plants, including plant height, stem development, flowering time, and flower bud formation [30–32]. In poplar, overexpressing *SPL16* and *SPL23* causes early growth cessation by coordinately suppressing the expression of *FLOWERING LOCUS T2* (*FT2*) and activating the expression of *BRANCHED1* orthologs (*BRC1.1* and *BRC1.2*) [33]. In maize, overexpressing *ZmSPL12* decreases the gibberellin (GA) level by directly inhibiting GA3-oxidase (*ZmGA3ox2*) transcription, which reduces plant height and increases lodging resistance [34]. AtWRKY12, MdWRKY9, OsWRKY24, and OsWRKY21 are transcriptional repressors that regulate plant height and stem development [35–37]. In rice, *OsWRKY36* reduces the plant height by repressing GA signaling [38]. Additionally, WRKY TFs modulate the flowering time in *Arabidopsis* [39, 40]. WRKY63 promotes vernalization-induced flowering by directly activating the expression of the lncRNAs, *COOLAIR*, and *COLDAIR* [41]. AtWRKY12 and AtWRKY13 physically interact with DELLA proteins to regulate GA-mediated flowering [42]. However, the regulatory mechanisms of WRKYs during vegetative phase change in woody plants require further investigation.

Thus, this study showed that the vegetative phase change in apple contributes to age-dependent changes in plant growth, photosynthetic performance, hormone levels, and carbon distribution. RNA-seq analysis revealed that MdSDH1, MdGASA1, MdSPL1, and MdWRKY24 play critical roles in the transition from juvenile to adult phase. Further studies showed that *MdSDH1* is important in regulating sorbitol levels via the MdSPL1-MdWRKY24 module. Moreover, sorbitol and MdSPL1-MdWRKY24 module form a feedback loop that regulates the expression of the GA response gene, *MdGASA1*, thus modulating the transition of growth strategies during vegetative phase change in apple. Our results provide new insights into the regulatory mechanism by which sorbitol-mediated growth transitions in apple.

## Results

### Plant morphological and leaf physiological age-dependent changes during vegetative phase change in apple

**Figure 1:**
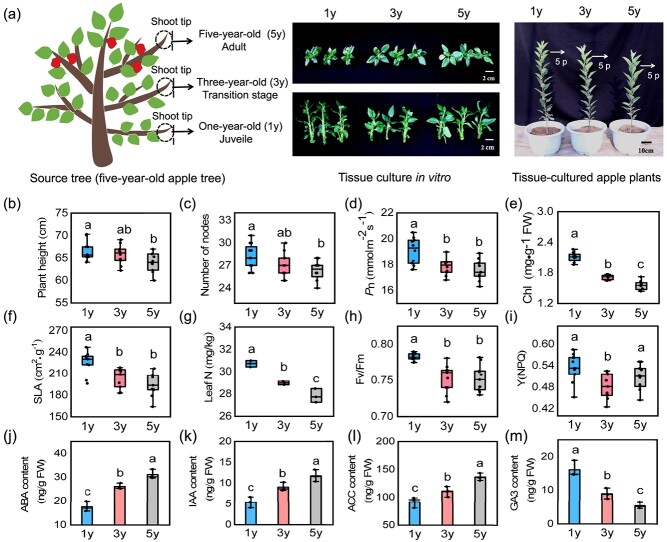
Morphological and leaf physiological changes during juvenile to adult vegetative phase in apple. **(a)** The shoot tips were collected from the 1-, 3-, and 5-year-old branch of the source tree and obtained tissue-cultured apple plants at juvenile, transition, and adult phase via *in vitro* tissue culture, and named 1y, 3y, and 5y, respectively. p represents leaf position. **(b)** The plant height, **(c)** internode number, **(d)** net photosynthesis rate (Pn), **(e)** chlorophyll content (Chl), **(f)** specific leaf area (SLA). Values represent means ± SD (*n* = 10). **(g)** leaf nitrogen (leaf N). Values represent means ± SD (*n* = 3). **(h)** Maximal photochemical efficiency (Fv/Fm) and **(i)** regulatory energy dissipation (Y(NPQ)) of 1y, 3y, and 5y apple plants. Values represent means ± SD (*n* = 11). **(j)** ABA content, **(k)** IAA content, **(l)** ACC content, and **(m)** GA3 content of 1y, 3y, and 5y apple plants. Values represent means ± SD (*n* = 3). Values with different letters are significantly different based on one-way ANOVA and Tukey’s test (*P* < 0.05).

Vegetative phase changes in apple can be divided into three stages (juvenile, transition, and adult) (Fig. 1a). In a previous study, we obtained tissue-cultured apple plants at juvenile, transition, and adult phase and designated 1y, 3y, and 5y. Results showed that the dynamic changes of leaf size, abaxial trichome, epidermal cell size, stomatal density, SLA, and miR156 level in tissue-cultured apple plants (1y, 3y, and 5y) were consistent with those in the source tree (different stages) [43]. Moreover, we revealed the regulatory mechanism of CK-mediated changes in leaf size during vegetative phase change [43]. To further investigate how vegetative phase change contributes to the growth strategies changes in apple, we compared the plant morphology across 1y, 3y, and 5y apple plants. The results showed an obvious decrease in plant height and internode number from 1y to 5y plants, suggesting a shift from the quick to the slow-growth strategy during the vegetative phase change (Fig. 1a–c). The net photosynthetic rate (Pn), chlorophyll (Chl) content, SLA, leaf nitrogen (leaf N), maximal photochemical efficiency (Fv/Fm), and regulatory energy dissipation (Y(NPQ)) continuously decreased from 1y to 5y plants (Fig. 1d–i), consistent with the phenotype of the 1y, 3y, and 5y apple plants.

Moreover, the contents of four hormones (abscisic acid, ABA; gibberellin 3, GA3; auxin, IAA; 1-aminocyclopropanecarboxylic acid, ACC) in 1y, 3y, and 5y apple plants were measured. The content of ABA, IAA, and ACC gradually increased from 1y to 5y plants (Fig. 1j–l), suggesting that they play positive roles in the transition from juvenile to adult phase in apple. In contrast, the GA3 content gradually decreased from 1y to 5y plants. The GA3 content of 1y plants were 1.89-fold and 3.08-fold higher than in 3y and 5y plants, respectively (Fig. 1m).

### Leaf carbon distribution age-dependent changes during vegetative phase change in apple

**Figure 2:**
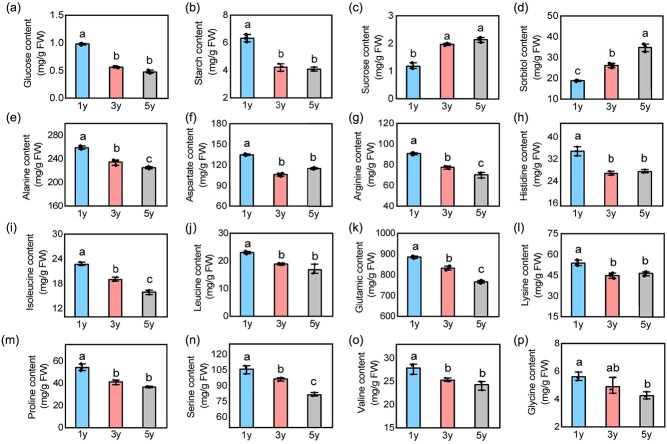
Sugar and amino acid contents during juvenile to adult vegetative phase in apple. **(a)** The content of glucose, **(b)** starch, **(c)** sucrose, **(d)** sorbitol, **(e)** alanine, **(f)** aspartate, **(g)** arginine, **(h)** histidine, **(i)** isoleucine, **(j)** leucine, **(k)** glutamic, **(l)** lysine, **(m)** proline, **(n)** serine, **(o)** valine, and **(p)** glycine in 1y, 3y, and 5y apple plants. Values represent means ± SD (*n* = 3). Values with different letters are significantly different based on one-way ANOVA and Tukey’s test (*P* < 0.05).

We further measured the contents of sugars and amino acids in 1y, 3y, and 5y apple plants by targeted metabolomics. The contents of glucose, starch, and 12 amino acids (alanine, aspartate, arginine, histidine, isoleucine, leucine, glutamate, lysine, proline, serine, valine, and glycine) gradually decreased from 1y to 5y plants (Fig. 2a, b, e–p). On the contrary, the contents of sucrose and sorbitol gradually increased from 1y to 5y plants (Fig. 2c and d), suggesting that they play positive roles in the transition from juvenile to adult phase in apple. These results demonstrate that the photosynthetic products in 1y plants may be used for growth but accumulated as sugars in 5y plants. Moreover, 60–80% of the photosynthates in apple leaves were sorbitol [44], with approximately 19-fold higher contents than sucrose (Fig. 2c and d), suggesting that sorbitol is the major carbon source during apple growth. Thus, sorbitol might participate in the transition from the quick to the slow-growth strategy in apple.

### RNA-seq data shows that *MdSDH1* is related to vegetative phase change

**Figure 3:**
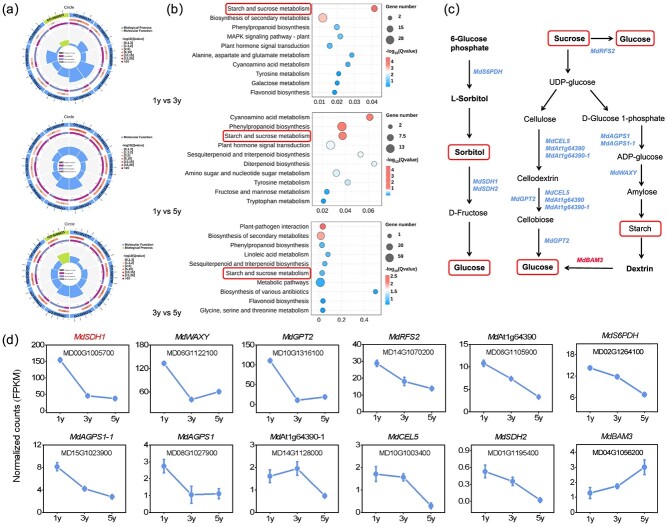
RNA-seq identification of *MdSDH1* from juvenile to adult vegetative phase in apple. **(a)** Gene Ontology (GO) enrichment of DEGs. **(b)** KEGG pathway enrichment analysis of DEGs in the three pairwise comparisons. **(c)** Proposed metabolism of photosynthates (sugars) in apple plants. **(d)** The line chart represents the DEGs (FPKM) in 1y, 3y, and 5y apple plants.

To identify genes associated with age-dependent changes in growth strategy during vegetative phase change, we performed an RNA-Seq analysis in the top fifth or sixth fully expanded leaves of 1y, 3y, and 5y apple plants. The three RNA-Seq biological replicates had high Pearson correlation analysis, indicating the reliability of sequencing data (Fig. S1, see online supplementary material). By comparing the gene expression levels, 211, 648, and 560 differentially expressed genes (DEGs) were identified in the comparisons of 1y vs. 3y, 1y vs. 5y, and 3y vs. 5y, respectively (Fig. S2a, see online supplementary material). The DEGs enriched the following GO terms: oxidation–reduction process (GO: 0055114), carbohydrate binding (GO: 0030246), energy reserve metabolic process (GO: 0005975), carbohydrate metabolic process (GO: 0005975), and other biological processes (Fig. 3a). Further, the DEGs in 1y vs 3y vs 5y comparisons significantly enriched the following KEGG pathways: carbohydrate metabolism, amino acid metabolism, and biosynthesis of other secondary metabolites (Fig. S2b, see online supplementary material), including ‘starch and sucrose metabolism’, ‘phenylpropanoid biosynthesis’, and ‘tyrosine metabolism’ (Fig. 3b).

Twelve DEGs (*MdSDH1*, *MdSDH2*, *MdWAXY*, *MdGPT2*, *At1g64390*, *MdS6PDH*, *MdRFS2*, *MdAGPS1*, *MdAGPS1–1*, *At1g64390–1*, *MdCEL5*, and *MdBAM3*) were involved in carbon assimilation metabolism pathway, and all showed an age-related expression pattern (Fig. 3c and d). Metabolism pathway showed that sorbitol dehydrogenase (MdSDH1/2) oxidized sorbitol into fructose (Fig. 3c), and it showed an age-dependent expression pattern (Fig. 3d). The expression gradually decreased from 1y to 5y plants, causing sorbitol to accumulate during the vegetative phase transition (Figs 2dand 3d). Obviously, the expression pattern and FPKM of *MdSDH1* were significantly higher than *MdSDH2*; thus, *MdSDH1* was selected for subsequent experiments (Fig. 3d)*.* As previously mentioned, sorbitol levels represent the carbon and energy status in apple [26]. Therefore, we hypothesize that MdSDH1 may be a master regulator controlling the shift from quick to slow-growth strategy in apple.

### MdSDH1 regulates plant growth by altering endogenous GA3 levels in apple

**Figure 4:**
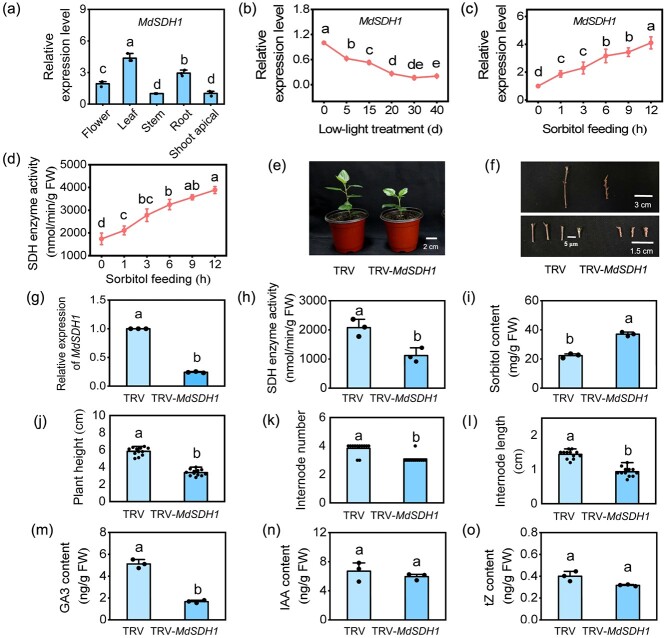
Silencing *MdSDH1* inhibits apple plant growth. **(a)** Relative expression levels of *MdSDH1* in different apple tissues. **(b)** Expression analysis of *MdSDH1* in apple leaves under low-light treatment. **(c)** Expression analysis of *MdSDH1* and **(d)** SDH enzyme activity in apple leaves under 50 uM sorbitol feeding. **(e)** and **(f)** Phenotypes of TRV-*MdSDH1* and TRV apple plants. **(g)** Relative expression level of *MdSDH1*, **(h)** SDH enzyme activity, **(i)** sorbitol content, **(j)** plant height, **(k)** internode number, **(l)** internode length, **(m)** GA3 content, **(n)** IAA content, and **(o)** tZ content in TRV-*MdSDH1* and TRV plants. Values represent means ± SD (*n* = 3). Values with different letters are significantly different based on one-way ANOVA and Tukey’s test (P < 0.05).

The full-length coding sequence of *MdSDH1* is 1107 bp and encodes a protein with 368 amino acids. Reverse transcription-quantitative PCR (RT-qPCR) showed that *MdSDH1* is expressed in all tested apple tissues, but the expression is highest in apple leaves (Fig. 4a). An expression analysis revealed that low-light treatment significantly down-regulated *MdSDH1* expression (Fig. 4b), indicating that *MdSDH1* is responsive to environmental signals in apple plants. We further fed the youngest fully expanded leaves from 30-day-old seedlings with 50 mM sorbitol for 12 h and examined *MdSDH1* transcript levels and SDH enzyme activity at different time points. Exogenous sorbitol strongly induced *MdSDH1* transcript levels and SDH enzyme activity (Fig. 4c and d), suggesting that MdSDH1 is a key enzyme for sorbitol catabolism in apple.

We constructed silencing vectors in apple plants via virus-induced gene silencing (VIGS) assays to analyse the function of *MdSDH1* in apple. RT-qPCR analysis showed that *MdSDH1* transcripts in TRV-*MdSDH1* plants were significantly reduced by 75% compared with TRV plants (Fig. 4g). Additionally, *MdSDH1* silencing significantly reduced SDH enzyme activity and promoted sorbitol accumulation in the TRV-*MdSDH1* apple plants (Fig. 4h and i), demonstrating that the SDH enzyme governs sorbitol catabolism. Phenotypic analysis revealed that TRV plants were taller than TRV-*MdSDH1* plants after 30 days (Fig. 4e, f, and j). At the same time, the numbers and lengths of internodes, which primarily determine the plant height, were significantly lower in TRV-*MdSDH1* plants than in TRV plants (Fig. 4 k and l). These results demonstrate that silencing *MdSDH1* inhibits apple plant growth. Besides, exogenous sorbitol application significantly reduced the plant height and internode length (Fig. S3a–d, see online supplementary material), suggesting that sorbitol accumulation significantly inhibits apple plant growth. This result was consistent with the previous finding that the growth ability in 1y apple plants was stronger than that 5y apple plants when sorbitol accumulated in 5y apple plants (Figs 1b and 2d).

Various endogenous hormones affect plant growth; we thus measured the GA3, IAA, and cytokinin trans-zeatin (tZ) levels in TRV and TRV-*MdSDH1* plants. Compared with TRV plants, the contents of IAA and tZ were insignificantly different, while the GA3 content was significantly lower in TRV-*MdSDH1* (Fig. 4m–o). To further confirm whether the TRV-*MdSDH1* phenotype is caused by GA3 deficiency, we treated TRV-*MdSDH1* plant with 10 μM exogenous GA3. The GA3 application promoted the growth of TRV-*MdSDH1* apple plants (Fig. S4a–d, see online supplementary material), indicating that *MdSDH1* silencing decreased the GA3 content, thereby repressing the growth of apple.

### *MdSDH1* is directly regulated by MdSPL1 and MdWRKY24

**Figure 5:**
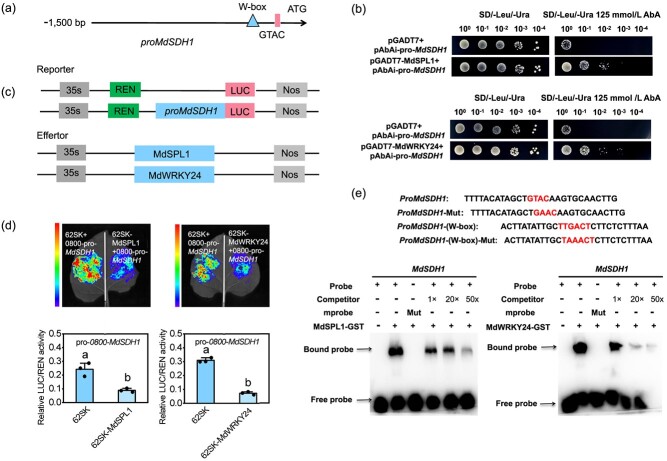
MdSPL1 and MdWRKY24 bind directly to *MdSDH1* promoter*.***(a)** Schematic diagram of the *MdSDH1* promoter. **(b)** Y1H assay identify the binding ability of MdSPL1 and MdWRKY24 on *MdSDH1* promoter. **(c)** Schematic diagram of reporter and effector constructs. **(d)** The images of luciferase and relative LUC/REN activities between *MdSDH1* promoter and MdSPL1 and MdWRKY24. **(e)** EMSA assay of MdSPL1 and MdWRKY24 proteins binding to *MdSDH1* promoter. Mut, mutant probes. Values represent means ± SD (*n* = 3). Values with different letters are significantly different based on one-way ANOVA and Tukey’s test (*P* < 0.05).

To further study the mechanism for MdSDH1 during vegetative phase change, we used the *MdSDH1* promoter as the bait in a yeast-one hybrid (Y1H) system to screen apple cDNA library. The results showed that a SBP (SPL) TF, (MdSPL1), and a WRKY TF (MdWRKY24) binds to the *MdSDH1* promoter in yeast cells (Fig. 5a and b). Previous studies showed that SPLs and WRKY TFs are important in regulating plant growth [34, 37, 45, 46]. In this study, an analysis by RT-qPCR showed that the transcript levels of MdSPL1 and MdWRKY24 were linearly increasing from 1y to 5y plants (Fig. S5a and b, see online supplementary material), suggesting that they are age-dependent TFs. Our previous studied demonstrated that CK regulates age-mediated changes in leaf size through the mdm-miR156a/MdSPL14 module regulate in apple [43]. Sequence complementarity analysis showed that no binding region of mdm-miR156a was detected on *MdSPL1* mRNA (Fig. S6a, see online supplementary material). Moreover, no decrease in the expression of *MdSPL1* was detected when the mdm-miR156a was overexpressed in apple leaves via transient infiltration (Fig. S6b, see online supplementary material). To further verify the mdm-miR156a on MdSPL1 activity, a dual luciferase-based miRNA sensor assay was conducted in tobacco leaves. There was no decrease in fluorescence signal and the relative Luciferase/Renilla (LUC/REN) activity with the co-expression of mdm-miR156a and *MdSPL14* compared with the control group (Fig. S6c and d, see online supplementary material). Therefore, these results demonstrate that mdm-miR156a is unable to directly target MdSPL1 in apple.

The dual-luciferase (LUC) assay revealed that expressing MdSPL1 and MdWRKY24 in *Nicotiana benthamiana* leaves significantly decreased the relative LUC/REN activity driven by the *MdSDH1* promoter (Fig. 5c–d), suggesting that MdSPL1 and MdWRKY24 repressed *MdSDH1* transcription*.* The electrophoretic mobility shift assay (EMSA) showed that MdSPL1 and MdWRKY24 can bind directly to the GTAC sites and W-box in the *MdSDH1* promoter, respectively (Fig. 5e). The ability of MdSPL1 and MdWRKY24 to bind the GTAC sites and W-box in the *MdSDH1* promoter region gradually decreased as the amount of competitor probes increased. After the core base of the W-box and GTAC sites mutated, MdWRKY24 and MdSPL1 cannot bind to the mutant probe (Fig. 5e). These findings reveal that MdSPL1 and MdWRKY24 function as *MdSDH1* transcriptional inhibitors.

### The MdSPL1-MdWRKY24 module promotes sorbitol accumulation by repressing *MdSDH1* expression

**Figure 6:**
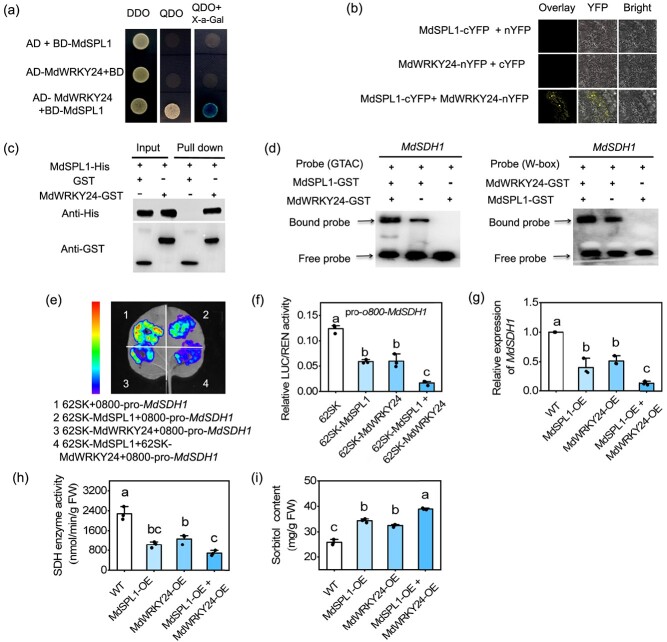
MdSPL1 interacts with MdWRKY24 to coordinately repress *MdSDH1* expression. **(a)** A Y2H assay shows the interaction of MdSPL1 and MdWRKY24 in yeast cells. **(b)** BiFC assay shows that the interaction of MdSPL1 and MdWRKY24 in *Nicotiana benthamiana* leaves. **(c)** Pull-down assay shows that the interaction of MdSPL1 and MdWRKY24 in *in vitro.***(d)** EMSA assays show that the MdSPL1–MdWRKY24 interaction enhances the binding of MdSPL1 and MdWRKY24 to the 5′ biotin probes. **(e)** and **(f)** LUC assays show that the MdSPL1–MdWRKY24 interaction enhances the repression of MdSPL1 and MdWRKY24 on *MdSDH1*. **(g)** Expression levels of *MdSDH1*, **(h)** sorbitol content, and **(i)** SDH enzyme activity in apple leaves with the overexpression of *MdSPL1*, *MdWRKY24*, and *MdSPL1* + *MdWRKY24*. Values represent means ± SD (*n* = 3). Values with different letters are significantly different based on one-way ANOVA and Tukey’s test (*P* < 0.05).

An assessment of whether MdSPL1 physically interacted with MdWRKY24 by yeast-two hybrid (Y2H) assay revealed that the yeast strains with co-expressed MdSPL1 and MdWRKY24 had normal growth on the -T/−L/-H/−A media (Fig. 6a). To further validate the interaction of the two proteins, an *in vivo* bimolecular fluorescence complementation (BiFC) assay was conducted. The result showed that a yellow fluorescence signal was observed in the nucleus and cell membranes when MdSPL1-cYFP and MdWRKY24-nYFP were co-transformed in *N. benthamiana* leaves (Fig. 6b). A pull-down assay using MdSPL1-His and MdWRKY24-GSH protein revealed that MdSPL1 and MdWRKY24 interact *in vitro* (Fig. 6c). Furthermore, EMSA assays were performed to determine the consequence of the MdSPL1–MdWRKY24 interaction on the transcriptional regulation of *MdSDH1*. The addition of MdSPL1 protein and MdWRKY24 protein significantly enhanced the binding of both proteins to the biotin probes, respectively (Fig. 6d), indicating that the interaction between MdSPL1 and MdWRK24 increase the binding ability of both MdSPL1 and MdWRK24 proteins on *MdSDH1* promoter. A LUC assay on *N. benthamiana* leaves further showed that the MdSPL1 and MdWRKY24 co-expression significantly repressed the *MdSDH1 _pro_: LUC* expression than the effect of MdSPL1 or MdWRKY24 alone (Fig. 6e and f). Therefore, there findings indicate that MdSPL1 interacts with MdWRKY24 and enhance the inhibition of *MdSDH1* by both MdSPL1 and MdWRKY24.

To confirm the role of MdSPL1 and MdWRKY24 in regulating endogenous sorbitol levels, *MdSPL1-OE*, *MdWRKY24-OE*, and the *MdSPL1-OE* + *MdWRKY24-OE* combination were overexpressed in apple leaves via vacuum infiltration. RT-qPCR analysis showed that overexpression of *MdSPL1* and *MdWRKY24* in apple leaves significantly reduced the transcript levels of *MdSDH1* (Fig. S7a and b, see online supplementary material; Fig. 6g). Obviously, co-expressing MdSPL1-OE and MdWRKY24-OE significantly reduced SDH enzyme activity, leading to a higher sorbitol content than in MdSPL1-OE or MdWRKY24-OE alone (Fig. 6h and i). These results demonstrate that the MdSPL1-MdWRKY24 module positively regulates sorbitol levels by repressing *MdSDH1* expression in apple.

### Sorbitol inhibits *MdGASA1* expression via the MdSPL1-MdWRKY24 module

The above results support the viewpoint that sorbitol represses plant growth by affecting the GA3 level, but the role of GA signaling involved in plant growth during the juvenile to adult vegetative phase remains yet to have been studied. RNA-seq data further revealed a GA-responsive gene Gibberellic Acid-Stimulated *Arabidopsis* (*MdGASA1*) gene that exhibited age-dependent expression patterns in apple, with high expression in 1y plants, which gradually decreased during the vegetative phase change (Fig. S8a, see online supplementary material). RT-qPCR analysis showed that GA3 treatment strongly up-regulated *MdGASA1* expression, which was down-regulated by sorbitol feeding (Fig. S8b and c, see online supplementary material), indicating that *MdGASA1* is a positive regulator of the GA signaling but is a negative regulator of sorbitol signaling in apple.

**Figure 7:**
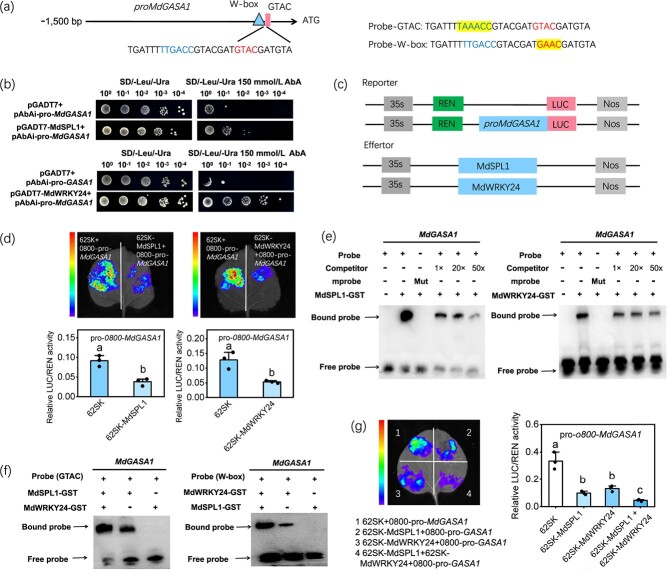
MdSPL1 and MdWRKY24 coordinately repress *MdGASA1* expression. **(a)** Schematic diagram of the *MdGASA1* promoter. **(b)** Y1H assays identify the binding ability of MdSPL1 and MdWRKY24 on *MdGASA1* promoter. **(c)** Schematic diagram of reporter and effector constructs. **(d)** The images of luciferase and relative LUC/REN activities between *MdGASA1* promoter and MdSPL1 and MdWRKY24. **(e)** EMSA assays of MdSPL1 and MdWRKY24 proteins binding to *MdGASA1* promoter. Mut, mutant probes. **(f)** EMSA assays show that the MdSPL1–MdWRKY24 interaction enhances the binding of MdSPL1 and MdWRKY24 to the 5′ biotin probes. **(g)** LUC assays show that the MdSPL1–MdWRKY24 interaction enhances the repression of MdSPL1 and MdWRKY24 on *MdGASA1.* Values represent means ± SD (*n* = 3). Values with different letters are significantly different based on one-way ANOVA and Tukey’s test (*P* < 0.05).

To identify whether *MdGASA1* is directly regulated by MdSPL1 and MdWRKY24, Y1H, LUC and EMSA assays were performed. Y1H assays showed that yeast cells that co-expressed pro-*MdGASA1* and pGADT7-MdSPL1/pGADT7-MdWRKY24 grew normally on -Leu/-Ura media with 150 mmol/L of Aureobasidin (AbA) (Fig. 7b), suggesting that MdSPL1 and MdWRKY24 can bind directly to the *MdGASA1* promoter. LUC assay revealed that expressing MdSPL1 and MdWRKY24 in *N. benthamiana* leaves significantly decreased the relative LUC/REN activity driven by the *MdGASA1* promoter (Fig. 7c and d), suggesting that MdSPL1 and MdWRKY24 repress *MdGASA1* transcriptional activity*.* EMSA further showed that MdSPL1 and MdWRKY24 can bind directly to the GTAC sites and W-box in the *MdGASA1* promoter *in vitro*, respectively (Fig. 7e). These findings reveal that MdSPL1 and MdWRKY24 function as *MdGASA1* transcriptional inhibitors, which was consistent with the previous result that GA3 gradually decreases during vegetative phase change (Fig. 1m).

Furthermore, EMSA assays were performed to determine the consequence of the MdSPL1–MdWRKY24 interaction on the transcriptional regulation of *MdGASA1*. The addition of MdSPL1 protein and MdWRKY24 protein significantly enhanced the binding of both proteins to the biotin probes, respectively (Fig. 7f), indicating that the interaction between MdSPL1 and MdWRK24 increase the binding ability of both MdSPL1 and MdWRK24 proteins on *MdGASA1* promoter. LUC assay on *N. benthamiana* leaves further confirmed that the MdSPL1 and MdWRKY24 co-expression significantly decreased the *MdGASA1 _pro_: LUC* expression than the effect of MdSPL1 or MdWRKY24 alone (Fig. 7g), suggesting that MdSPL1 interacts with MdWRKY24 and enhances the inhibition of *MdGASA1* by both MdSPL1 and MdWRKY24. These results demonstrate that MdSPL1 and MdWRKY24 coordinately repress *MdGASA1* transcription.

A previous study has shown that sorbitol plays a key signaling role in by regulating the expression of TFs, thereby controlling downstream target genes in apple [47]. Exogenous sorbitol application in apple leaves was conducted to determine whether sorbitol signaling is involved in *MdGASA1* transcription by affecting the MdSPL1-MdWRKY24 module. The results showed that exogenous sorbitol significantly induced *MdSPL1* and *MdWRKY24* transcript levels in apple leaves (Fig. S9a and b, see online supplementary material), indicating a feedback loop between sorbitol and the MdSPL1-MdWRKY24 module. These results indicate that sorbitol inhibits the expression of *MdGASA1* by activating MdSPL1 and MdWRKY24 in apple.

### Silencing *MdGASA1* decreased plant growth by affecting cell expansion in apple

**Figure 8:**
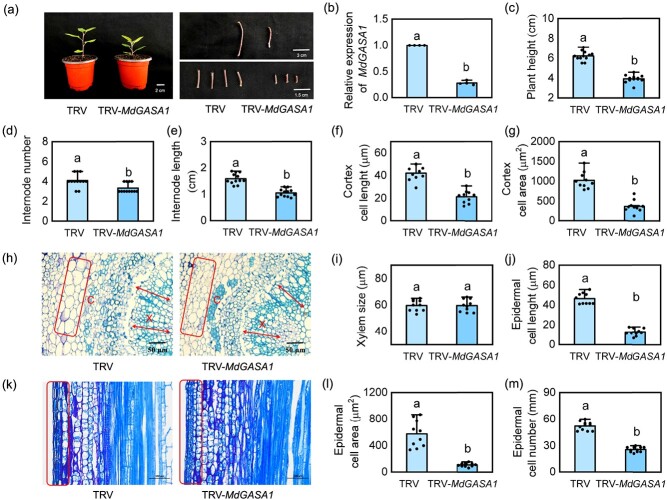
Silencing *MdGASA1* inhibits cell elongation in apple. **(a)** Phenotype of TRV-*MdGASA1* and TRV apple plants. **(b)** Relative expression levels of *MdGASA1*. Values represent means ± SD (*n* = 3). **(c)** Plant height, **(d)** internode number, and **(e)** internode length in TRV and TRV-*MdGASA1* plants. **(h)** Cross sections of the internodes. C: cambium, X: xylem. **(f)** Cortex cell length, **(g)** cortex cell area, and **(i)** xylem size based on the cross sections of TRV-*MdGASA1* and TRV plants. **(k)** Longitudinal sections of the internodes. **(j)** Epidermal cell length, **(l)** epidermal cell area, and **(m)** epidermal cell number based on the longitudinal sections of TRV-*MdGASA1* and TRV plants. Values represent means ± SD (*n* = 10). Values with different letters are significantly different based on one-way ANOVA and Tukey’s test (*P* < 0.05).

We generated *MdGASA1-*silenced plants through VIGS assay to validate the role of MdGASA1 in regulating plant growth. RT-qPCR analysis showed that *MdGASA1* transcripts in TRV- *MdGASA1* plants were significantly reduced by 72% compared with TRV plants (Fig. 8b). Phenotypic analysis showed that TRV plants had higher plant heights than the TRV-*MdGASA1* plants after 30 days (Fig. 8a and c). At the same time, the number and length of internodes were significantly higher in TRV than TRV-*MdGASA1* plants (Fig. 8d and e), implying that *MdGASA1* inhibits apple plant growth by shortening the internode length. Cytological observations of transverse stems showed that TRV plants had a significantly higher cortex cell length and cortex cell area than TRV-*MdGASA1* plants, but the xylem size was insignificantly different (Fig. 8f–i). Similarly, the longitudinal stem cells were larger in TRV plants than in TRV-*MdGASA1* plants, and the cell numbers were higher in TRV plants than in TRV-*MdGASA1* plants (Fig. 8j–m). These results demonstrate that silencing *MdGASA1* suppresses cell expansion along the longitudinal axis, shortening the internode length.

**Figure 9:**
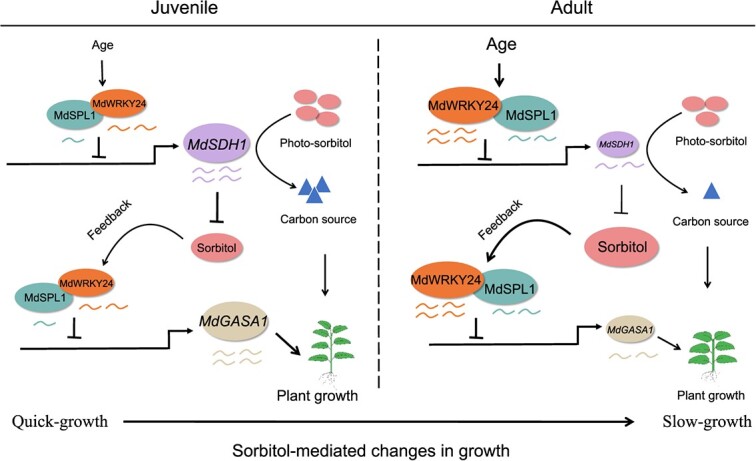
Proposed models illustrating how sorbitol mediates age-dependent changes in apple plant growth strategy through gibberellin signaling.

## Discussion

Vegetative phase change is a complex process in perennial woody plants [1–4]. Plants experience various age-dependent changes during this process, including morphological changes, photosynthetic traits, and growth strategies [1, 48–50]. However, changes in leaf sink-source balances, hormone dynamics, and abiotic stress responses are usually overlooked in apple plant research due to the limitations of the long juvenile phase. Thus, the relationship between these age-dependent changes and plant morphology in apple has remained unclear. This study obtained tissue-cultured apple plants to reveal the changes in plant growth, photosynthetic capacity, hormone levels, and carbon distribution during vegetative phase change. The study further provided detailed mechanistic evidence of the role of sorbitol in controlling the shifts from quick to slow-growth strategy in apple. The results highlight the function of sorbitol in connecting GA signaling pathways to regulate plant growth during juvenile to adult vegetative phase.

### Sorbitol is essential for the transition from quick to slow-growth strategy in apple

Sugars, including sucrose, glucose, and T6P, are a carbon source and key signaling molecules controlling the vegetative phase change in plants [5, 6, 51, 52]. Previous studies have shown that photosynthetic sucrose is not utilized directly; cytosolic invertase (CINV) irreversibly catalyzes its conversion to glucose and fructose, providing an essential carbon source for plant growth [53]. In *Arabidopsis*, sucrose-induced PAP1 TF increases the transcription of *SPL9* by directly binding to its promoter, which triggers the sucrose-mediated vegetative phase change through the miR156A/SPL9 module [54]. These results indicate that sugar accumulation in the leaves potentially functions as an age-dependent signal that regulates the downstream gene expression, contributing to vegetative phase change [5, 21]. Sorbitol is the primary photosynthetic product in Rosaceae fruit trees. In contrast, sucrose is the main photosynthate in the other plants [26, 27, 55] and is closely related to growth, development, and stress resistance [24, 26, 48].

In this study, sorbitol and *MdSDH1* exhibited an age-dependent pattern. *MdSDH1* was highly expressed in 1y plants, and sorbitol massively accumulated in 5y plants (Figs 2d and 3d). Moreover, the content of 12 amino acids gradually decreased during the vegetative phase change (Fig. 2e–p). Therefore, 1y plants possibly have strong sinks for protein synthesis and provide a steady carbon supply for energy production [4]. These results indicate that sorbitol mainly functions as a carbon source for the juvenile phase and a signal-modulating phase transition in the adult phase. Additionally, the *MdSDH1*-silenced phenotype of apple plants revealed that sorbitol accumulation suppresses growth (carbon consumption), consistent with the changes of growth in 1y, 3y, and 5y apple plants. Therefore, sorbitol is a candidate marker for vegetative phase change, and MdSDH1 is likely the key hub gene for regulating sorbitol-mediated shifts from quick to slow-growth strategy during juvenile to adult vegetative phase in apple.

In *Arabidopsis*, SPL10 regulates the age-mediated vegetative phase change by interacting with WRKY12 and WRKY13 [56], indicating that SPLs and WRKYs TF are critical in the aging pathway. Interestingly, several WRKY TFs function as negative regulators of plant height by decreasing brassinosteroid (BR) production or repressing the expression of cell elongation genes [31, 37, 38, 47]. The results of this study revealed that MdSPL1 and MdWRKY24 target *MdSDH1*, synergistically suppressing the expression of *MdSDH1* by binding to its promoter (Fig. 5). At the same time, overexpressing *MdSPL1* and *MdWRKY24* in apple leaves decreased the SDH enzyme activity and promoted sorbitol accumulation (Fig. 6h and i). These findings demonstrate that the age-mediated MdSPL1-MdWRKT24 module represses apple plant growth by promoting sorbitol accumulation. The exogenous sorbitol spraying test further confirmed this point (Fig. S3, see online supplementary material).

### *MdGASA1* is involved in sorbitol-mediated growth transition via the MdSPL1-MdWRKY24 module in apple

Sorbitol also functions as a signaling substance that regulates plant growth and development. During flower bud formation in loquat, sorbitol promotes hyperoside biosynthesis by activating the transcription of *EjERF12* and the MADS-box TF family gene, *EjCAL* [27]. In apple, sorbitol regulates downstream developmental genes by activating a key TF, MYB39L [47]. Suppressing *MdMYB39L* expression in apple pollen results in abnormal stamen development and reduces pollen tube growth, and this phenotype can be partially restored by exogenous sorbitol application during flower development [47]. Previous studies have shown that sorbitol controls sugar transport into the pollen tube by regulating the expression of pollen tubule transporter HT1.7, thus promoting pollen tube growth in apple [28]. In this study, sorbitol significantly activated the expression of two age-related TF genes, *MdSPL1* and *MdWRKY24*. MdSPL1 interacts with MdWRKY24 to synergistically suppress *MdSDH1* expression (Fig. 6; Fig. S9, see online supplementary material), suggesting that sorbitol-induced MdSPL1 and MdWRKY24 transcription via a feedback loop. Moreover, MdSPL1 and MdWRKY24 act synergistically to inhibit *MdGASA1* expression, thus reducing GA signaling responses (Fig. 7). A recent study revealed that overexpressing *OsWRKY36* results in dwarfness in wheat due to the increased *SLENDER RICE 1* (*SLR1*) transcription and the suppression of GA signaling [38]. These results provide evidence that sorbitol is a signal that regulates GA responses by activating age-related TFs during vegetative phase change.

Gibberellin is important in determining plant height [40, 41, 57]. The miR166 target gene THB14-LIKE controls plant height by directly repressing GA biosynthesis genes (*GmGA1* and *GmGA2*) expression and activating GA catabolic gene *GIBBERLLIN 2 OXIDASE 2* (*GmGA2ox2*) expression in soybean [58]. The GASA family genes are key downstream response genes in the GA signaling pathway, whose expression is strongly induced by GA3 and functions as a positive regulator for plant growth and development [59, 60]. In *Arabidopsis*, *AtGASA6* regulates seed germination and hypocotyl elongation by integrating GAs, ABA, and glucose signaling [61]. In soybean, GmGBP1 and GmGAMYB interaction induces the GA signal to increase plant height by activating the *GmSAUR* bound to the *GmGASA32* promoters [62]. Overexpressing *GmGASA32* promotes plant growth by interacting with GmCDC25 [63]. These results demonstrate that silencing *MdGASA1* represses apple plant growth by reducing the elongation of shoot stem cells (Fig. 8).

In conclusion, this study provided detailed phenotypic and physiological evidence demonstrating that sorbitol regulates age-dependent changes in growth strategies for apple through the MdSPL1-MdWRKY24 module during vegetative phase change. Therefore, we propose a model for sorbitol-mediated changes in growth strategies from the juvenile to the adult vegetative phase (Fig. 9). During juvenile to adult vegetative phase, the age-mediated MdSPL1-MdWRKY24 module facilitates sorbitol accumulation by repressing *MdSDH1* transcription, thus reducing the carbon source supply and increasing the sorbitol signal. Simultaneously, the increased sorbitol signaling promotes MdSPL1-MdWRKY24 expression via a feedback loop, thereby repressing *MdGASA1* transcription and decreasing apple plant growth in the adult phase.

## Materials and methods

### Plant materials and growth conditions

The F1 progeny of ‘Ambrosia × Honeycrisp’ were grown in a nutrient bowl and placed in solar greenhouses (25°C, 16/8 h light/dark) at Northwest A&F University, Yangling (34°20′ N, 108°24′ E), Shaanxi Province, China in 2015. In mid-March 2018, 16 plants with branches were selected and planted in fields to grow. In 2020, we selected one apple tree with a short juvenile period from 16 five-year-old apple trees and named as the source tree. In the source tree, 1-, 3-, and 5-year-old branch represents juvenile, transition, and adult phases, respectively. Thus, the shoot tips were collected from the 1-, 3-, and 5-year-old branch of the source tree and obtained tissue-cultured apple plants at juvenile, transition, and adult phase via *in vitro* tissue culture [64], and named 1y, 3y, and 5y, respectively (Fig. 1a). The tissue-cultured apple plants were cultured on rooting media for 40 days and transferred into plastic pots (6 × 6 cm) with matrix soil of organic substrate/perlite/vermiculite (3:1:1) and grown in a light incubator. After 50 days, the plants were transplanted into plastic pots (30 × 18 cm) filled with the soil/organic matter (v:v, 5:1) mixture and placed in a greenhouse to grow.

### Physiological measurements and morphological observation

Plant height measurements were taken from four-month-old apple plants. The top fifth or sixth fully expanded leaves were used for physiological measurements. Leaf N content was detected in the dried samples using an AA3 continuous flow analyzer (SEAL, Germany). After leaf area was measured using a leaf area scanner (Perfection V19, EPSON, Nagano, Japan), the samples were dried in an oven at 65°C until constant weight. The SLA was calculated using the following formula: SLA = dry weight/leaf area. Chlorophyll was extracted for 24 h using 80% acetone, and the chlorophyll content was determined using a UV-1800 spectrophotometer (Shimadzu, Kyoto, Japan) at 645 and 663 nm [65]. Photosynthetic parameters were monitored using a CIRAS-3 portable photosynthesis system (CIRAS, MA, USA). Leaves were placed in the chamber (18 mm diameter) at 25°C, 1000 μmol m^−2^ s^−1^ illumination, 500 μmol s^−1^ airflow rate, and 400 μmol mol^−1^ CO_2_. The chlorophyll (Chl) fluorescence parameters were determined using a Plant Chlorophyll fluorescence imaging system (Walz, Effeltrich, Germany) after 30 min of exposure to darkness. Histological analysis of stems as described by Yao *et al.* [66] and a BX63 light microscope (Olympus, Japan) was used to observe the cross sections. ImageJ software (https://imagej.net/software/imagej/) was used to measure the cell length and area.

### RNA extraction and RT-qPCR analysis

Total RNA was extracted from the top fifth or sixth fully expanded leaves of apple plants using a plant RNA extraction kit (FOREGENE 129710 Co., Ltd, Chengdu, China). RT-qPCR analysis was performed in a LightCycler 96 instrument. The primers used are shown in Table S1 (see online supplementary material).

### Determinations of free amino acids and sugars

The top fifth or sixth fully expanded leaves of apple plants were used for determination of amino acids and sugars. Amino acids were extracted as described previously by Huo *et al.* [67]. The sugar contents were measured using the gas chromatography–mass spectrometry (GC–MS) system equipped with a DB-5MS column (ISQ & TRACE ISQ, Thermo Fisher Scientific, MA, USA), as previously described by Hu *et al.* [68]. Further, the sorbitol content was measured using test kits from Suzhou Comin Biotechnology test kits following the manufacturer’s protocol.

### Measurements of the contents of phytohormones

The top fifth or sixth fully expanded leaves of apple plants were used for determination of hormones. Phytohormones were extracted as previously described by Jia *et al.* [43]. The phytohormone contents were measured using a QTRAP5500 HPLC-MS (AB SCIEX, DC, USA) after nitrogen blowing.

### Exogenous GA3 and sorbitol treatment

Briefly, 30-day-old 1y apple plants were grown in plastic 8 × 8 pots and were sprayed with 10 μM GA3. For exogenous sorbitol feeding, leaves from 30-day-old seedlings were fed with exogenous sorbitol as described previously [47]. The fully expanded leaves were collected at 0, 1, 3, 6, 9, and 12 h after treatment for RT-qPCR analysis. TRV-*MdSDH1* apple plants were sprayed with 10 μM GA3 and 100 μM sorbitol (PH102-X; Coolaber) every 2 days, and the controls were water-treated. The indicators were calculated after 30 d.

### Low-light treatment

30-day-old 1y apple plants were used for low-light treatment. The plants were placed in a light incubator (25°C, 16/8 h light/dark) for 40 days. The normal light condition is 180 μmol m^−2^ s^−1^ and low-light condition is 35 μmol m^−2^ s^−1^. The fully expanded leaves were collected at 0, 5, 15, 20, 30, and 40 days after treatment for RT-qPCR analysis.

### Transcriptome sequencing

The top fifth or sixth fully expanded leaves of 1y, 3y, and 5y apple plants were used for RNA-seq. The eukaryotic mRNA was enriched using Oligo (dT) beads. The variations were determined using the RSEM software. Gene Denovo Biotechnology Co (Guangzhou, China) conducted subsequent analyses.

### Y1H assay

The *MdSDH1* and *MdGASA1* promoter fragments (1500 bp) were fused into the pHIS2 vector, and the CDSs of *MdSPL1* and *MdWRKY24* were fused into the pGADT7 vector. The fusion vectors were transformed into the Y187 yeast. The monoclonal clone selected from the SD/-Leu medium was inoculated to SD/-Leu- medium supplemented with Aureobasidin A (AbA) to observe yeast growth.

### Y2H assay

The CDS region of *MdSPL1* was fused into pGBKT7, and the CDS region of *MdWRKY24* was fused into pGADT7. The fusion vectors were co-transformed into yeast strain Y2H Gold. The monoclonal clone growing on the DDO (SD/−Leu/−Trp) medium was inoculated to the selective QDC (SD/−Leu/−Trp/-His/−Ade) and QDC + X-α-Gal medium to observe yeast growth, respectively.

### Dual-luciferase reporter assay

The promoter sequences (1500 bp) of *MdSDH1* and *MdGASA1* were fused into the reporter pGreenII 0800-LUC vector, and the CDS regions of *MdSPL1* and *MdWRKY24* were fused into the effector pGreenII62-SK vector. Next, specified combinations of the recombinant plasmids were co-expression into *N. benthamiana* leaves. After 48–60 h of transformation, LUC fluorescence image was observed using an *in vivo* plant imaging system (PlantView100; Guangzhou Biolight Biotechnology Co., Ltd, Guangzhou, China). The relative LUC/REN activity was detected using a dual-luciferase reporter gene assay kit (Yeasen, Shanghai, China).

### EMSA

The MdSPL1-GST and MdWRKY24-GST recombinant vectors were transformed into *Escherichia coli* Rosseta (DE3), and the proteins were purified using GST beads (Beyotime, Shanghai, China). The EMSA assays were conducted using a Light Shift Chemiluminescent EMSA Kit (Thermo Fisher Scientific).

### BiFC assay

The CDS regions of *MdSPL1* and *MdWRKY24* were cloned into the pSPYCE and pSPYNE vectors, respectively. The recombinant vectors were transformed into *Agrobacterium tumefaciens* GV3101 and co-expressed in *N. benthamiana* leaves. The fluorescence was observed using a laser scanning microscope (TCS-SP8 SR; Leica) after 48–60 h of injection.

### Pull-down assay

The CDS regions of *MdSPL1* and *MdWRKY24* were inserted into pET-32a and pGEX-4T-1 vectors, respectively. The recombinant vectors were transformed into *E. coli* (Rosetta strain) for protein expression. The purified MdWRKY24-GST protein was incubated to anti-GST magnetic beads at 4°C for 12 h and then the purified protein MdSPL1-His was added and incubated at room temperature for 2 h. A western blot was performed using anti-GST (Beyotime, Shanghai, China) and anti-His antibodies (Yeasen, Shanghai, China), respectively.

### Virus-induced gene silencing

A specific 200 bp fragment of *MdSDH1* and 185 bp fragment *MdGASA1* were inserted into the pTRV2 vector, and the recombinant vectors were transformed into *A. tumefaciens* strain GV3101. The agrobacteria that were harbored contained the pTRV2-*MdMdSDH1* or pTRV2-*MdGASA1*, and pTRV1 were cultured to an OD_600_ of approximately 1.0. The VIGS assays were conducted as described by Zhu *et al.* [69].

### Statistical analyses

Origin software, version 2020 was used for statistical analysis. The data are shown as values represent means ± SD (standard deviation). The statistical significance was determined by one-way ANOVA, and *P* < 0.05 was considered statistically significant.

## Supplementary Material

Web_Material_uhae192

## Data Availability

The authors confirm that all experimental data are available and accessible via the main text and/or the supplemental data. Accession numbers: MdSDH1 (MD00G1005700), MdSPL1 (MD03G1230600), MdWRKY24 (MD11G1059400), MdGASA1 (MD02G1132400).
