# Supplementary material for: Sorbitol mediates age-dependent changes in apple plant growth strategy through gibberellin signaling
Source: Hortic Res. 2024 Jul 11;11(8):uhae192. doi: 10.1093/hr/uhae192 (PMC11322524; doi:10.1093/hr/uhae192)
Supplement: Web_Material_uhae192 [file web_material_uhae192.zip › Supplemental Figure.docx]

**Contents**

**Supplemental Figures 1 to 9**

**
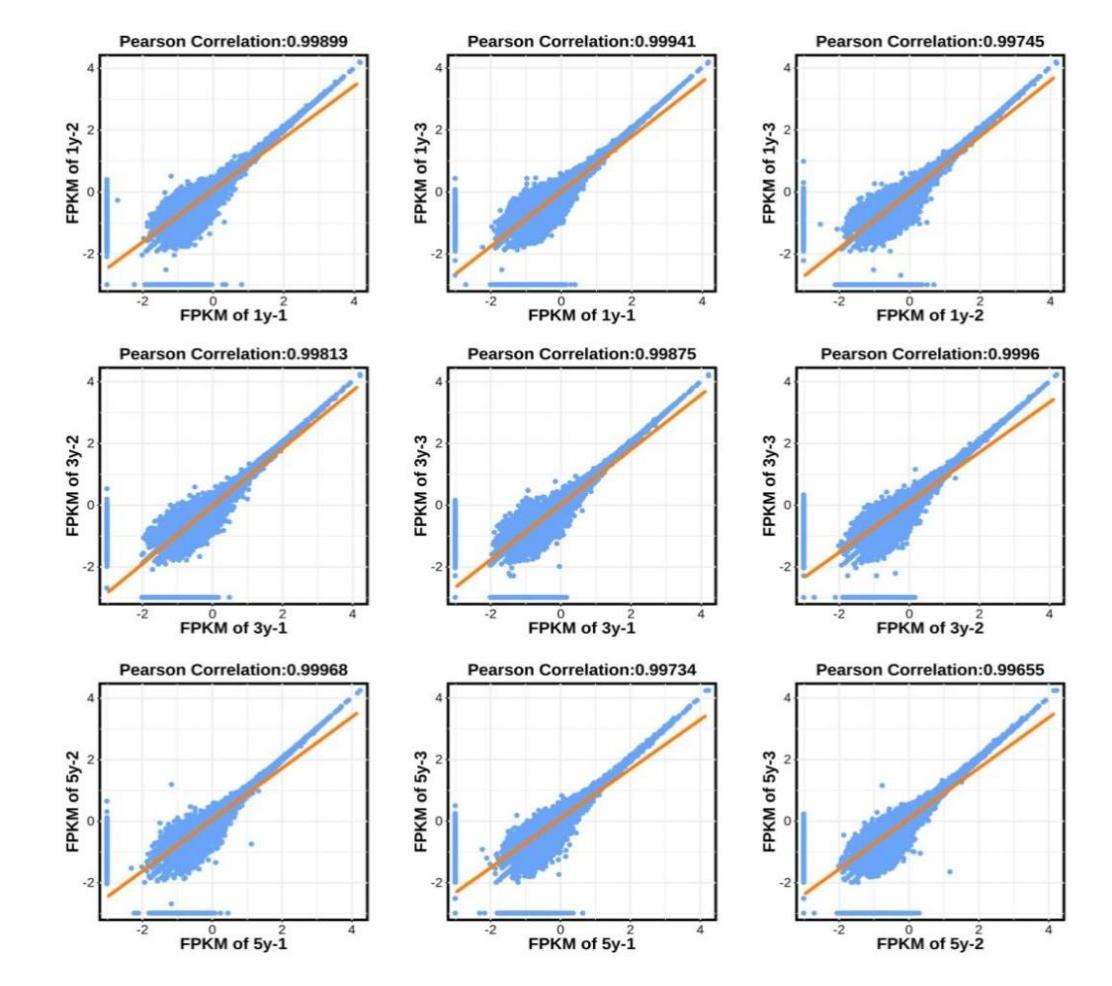
**

**Supplemental Figure S1 Pearson correlation analysis of the gene expression profile.**


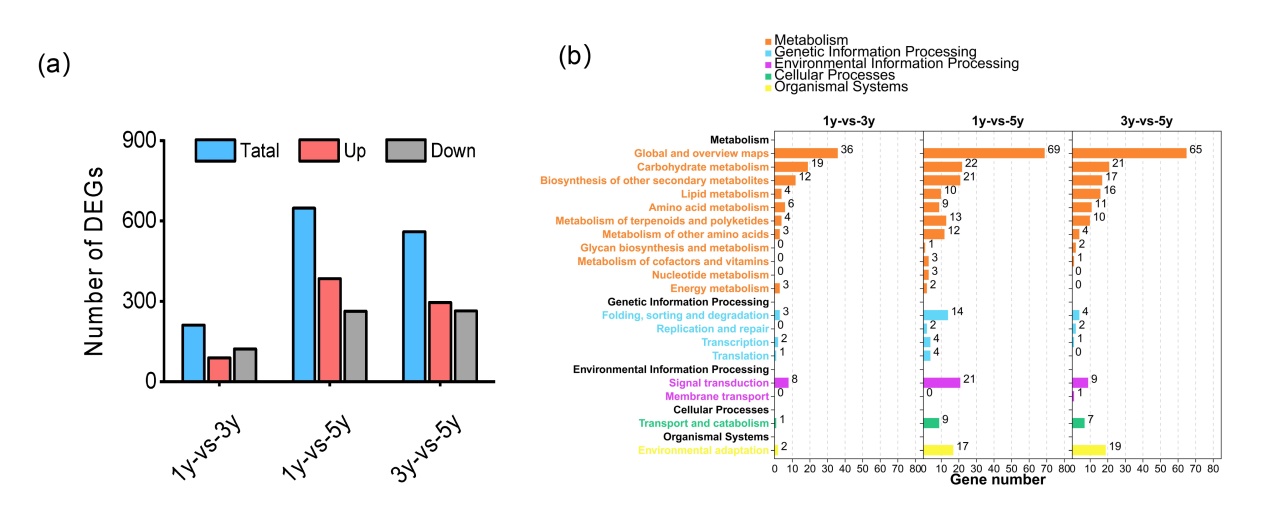


**Supplemental Figure S2 Transcriptome analysis of 1y, 3y, and 5y tissue-cultured apple plants. (a)** Bar diagram analysis of the DEGs in three pairwise comparisons (1y vs. 3y, 1y vs. 5y, and 3y vs. 5y, respectively). **(b)** KEGG enrichment analysis of the DEGs in 1y vs 3y vs 5y comparisons.

**
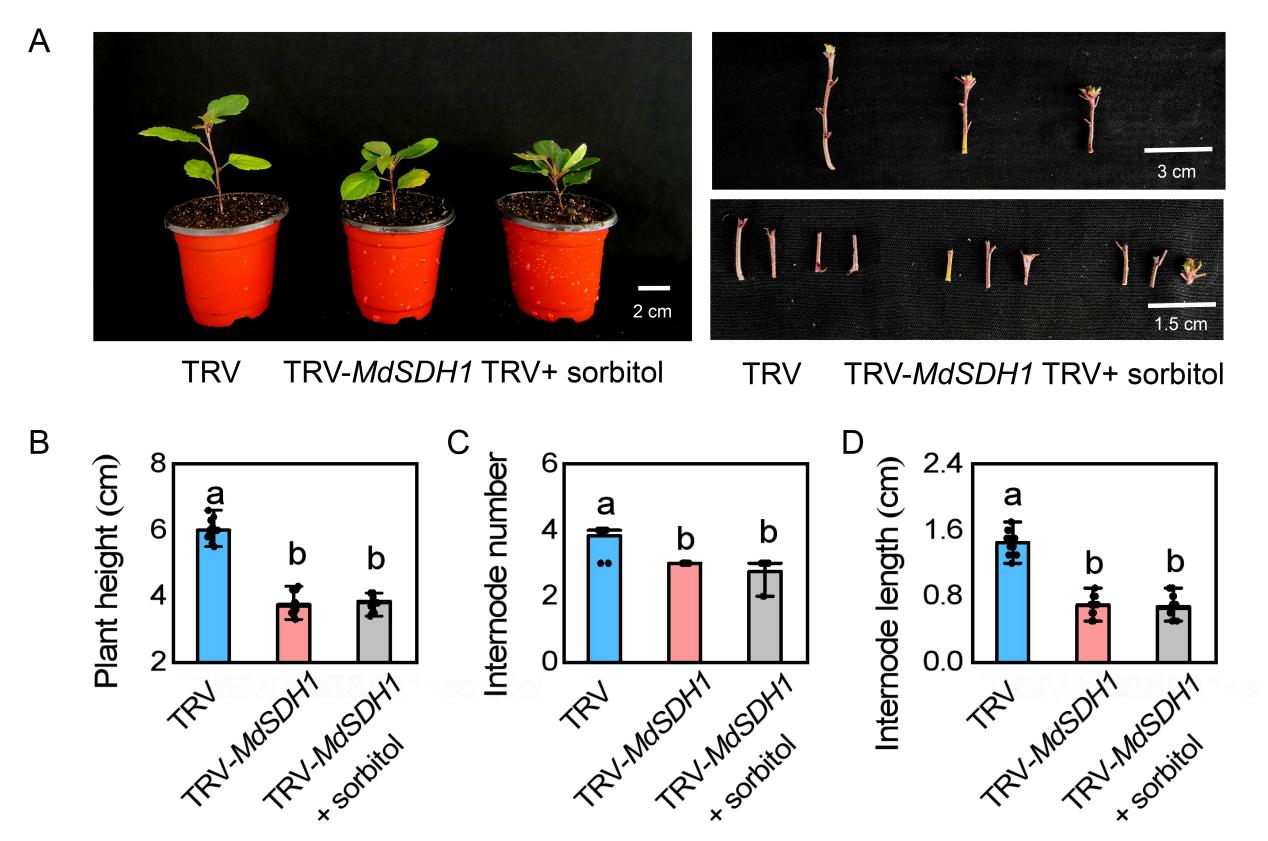
**

**Supplemental Figure S3** **Effect of exogenous sorbitol on TRV-*MdSDH1* apple plants. (a)** The phenotypes of TRV, TRV-*MdSDH1*, TRV + sorbitol apple plants. Control, spraying with water; + sorbitol, spraying with 100 μM sorbitol. **(b)** The plant height, **(c)** internode number, and **(d)** internode length in TRV, TRV-*MdSDH1*, and TRV + sorbitol apple plants. Values with different letters are significantly different according to one-way ANOVA followed by Tukey’s test (P < 0.05). All data ± SD (n=10).


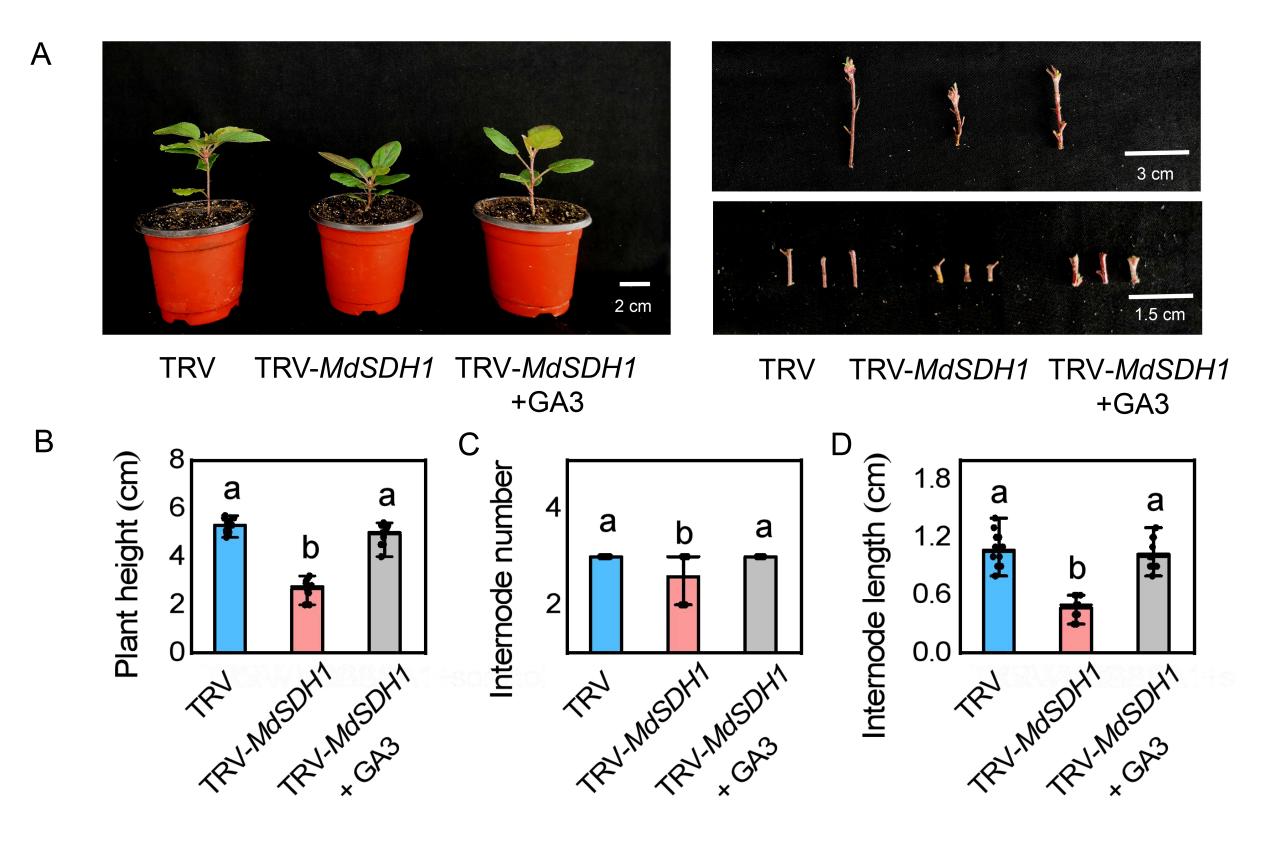


**Supplemental Figure S4 Effect of exogenous GA3 on TRV-*MdSDH1* apple plants. (a)** The phenotypes of TRV, TRV-MdSDH1, TRV + GA3 apple plants. Control, spraying with water; + GA3, spraying with 10 μM GA3. **(b)** The plant height, **(c)** internode number, and **(d)** internode length in TRV, TRV-*MdSDH1*, and TRV + GA3 apple plants. Values with different letters are significantly different according to one-way ANOVA followed by Tukey’s test (P < 0.05). All data ± SD (n=10).


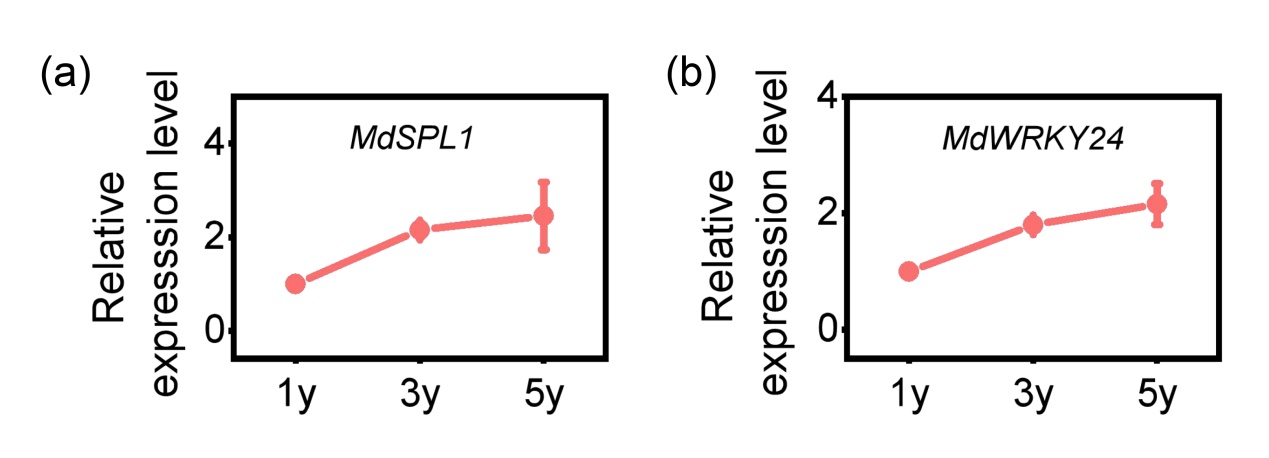


**Supplemental Figure S5 Relative expression levels of *MdSPL1* and *MdWRKY24* in 1y, 3y, and 5y tissue-cultured apple plants.** Values with different letters are significantly different according to one-way ANOVA followed by Tukey’s test (P < 0.05). Data are means ± SD (n=3).


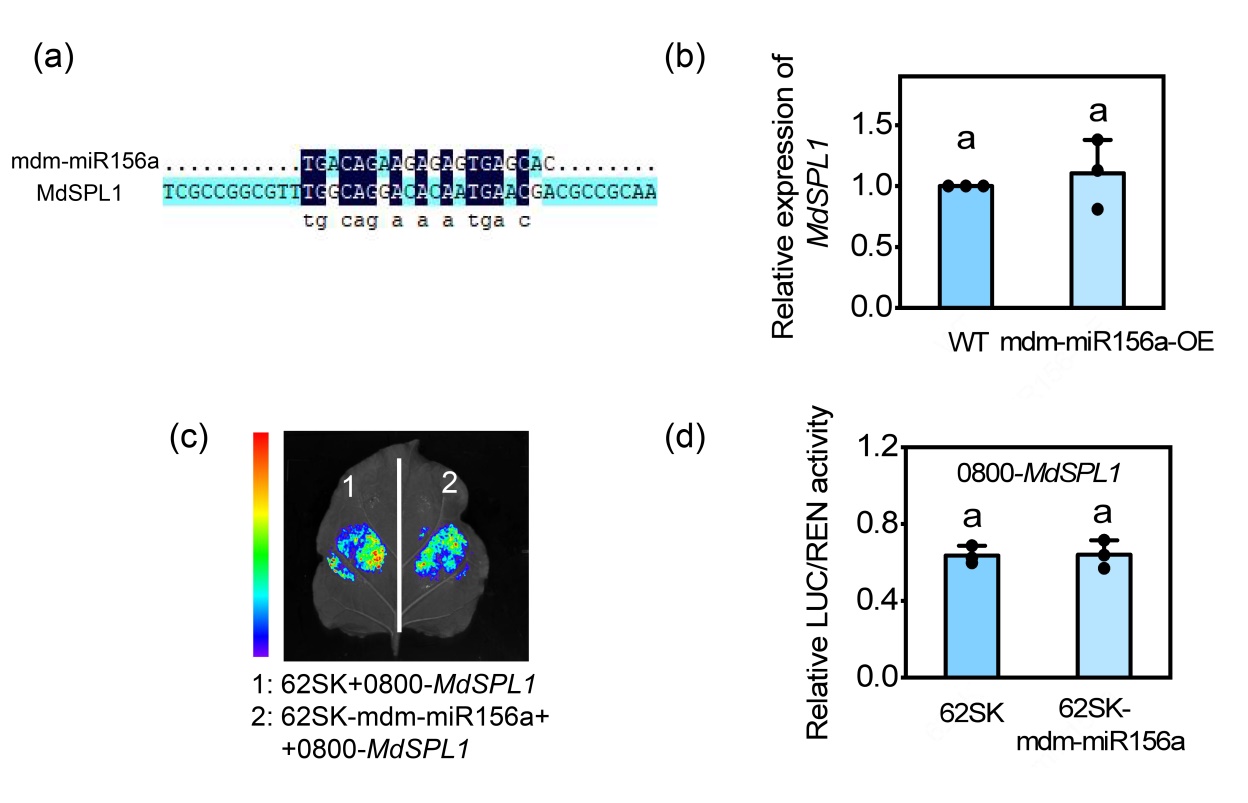


**Supplemental Figure S6 *MdSPL1* is not the direct target of mdm-miR156a.** (a) Sequence alignment of mdm-miR156a and MdSPL14. (b) The relative expression levels of *MdSPL1* in mdm-miR156-OE leaves. (c-d) A LUC assay shows the suppression of mdm-miR156a on *MdSPL14* in *N. benthamiana*. Values with different letters are significantly different according to one-way ANOVA followed by Tukey’s test (P < 0.05). All data ± SD (n=3).


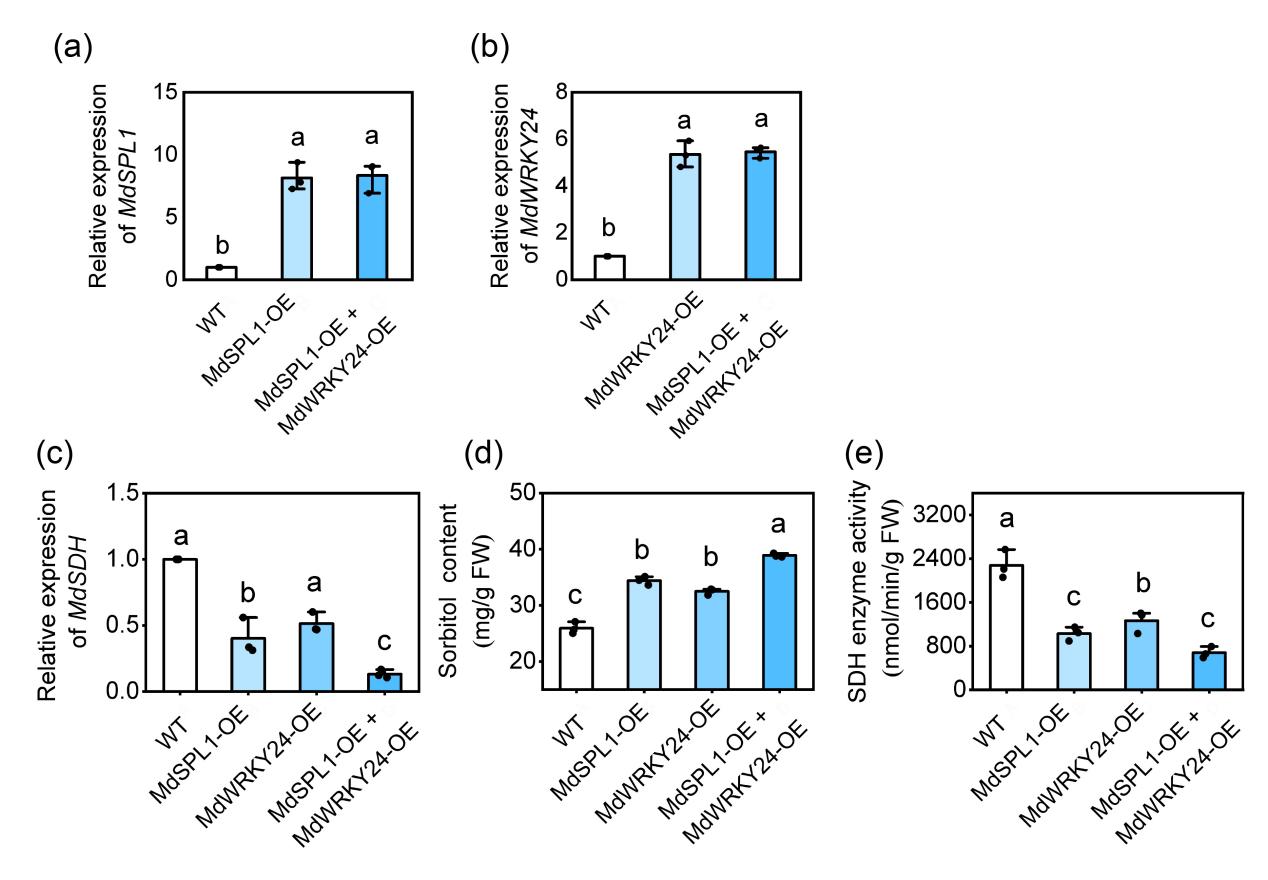


**Supplemental Figure S7** **Overexpression of *MdSPL1*, *MdWRKY24*, and *MdSPL*+*MdWRKY24* via agroinfiltration in apple leaves. (a)** The relative expression levels of *MdSPL1* in response to MdSPL1-OE, MdWRKY24-OE, and MdSPL1-OE+MdWRKY24-OE via agroinfiltration to leaves, with empty vector as control. **(b)** The relative expression levels of MdWRKY24. Values with different letters are significantly different according to one-way ANOVA followed by Tukey’s test (P < 0.05).

All data ± SD (n=3).


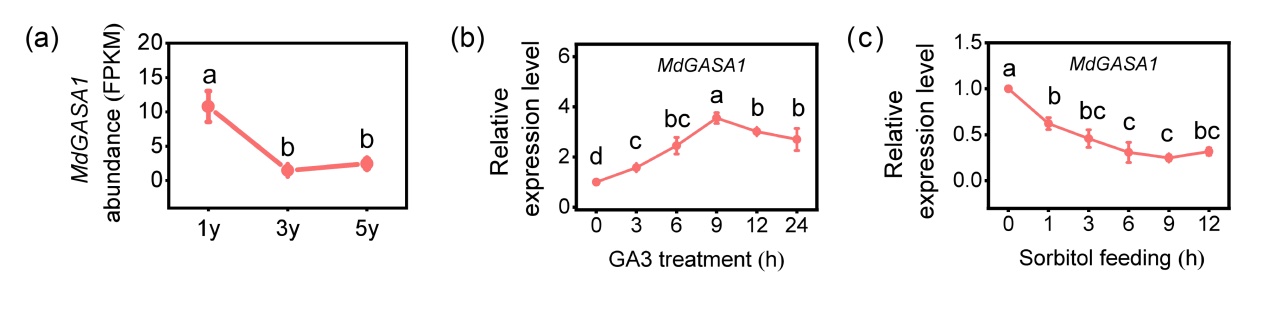


**Supplemental Figure S8 Expression of *MdGASA1* in apple leaves following GA3 and sorbitol treatment.** **(a)** Expression patterns of *MdGASA1* for 1y, 3y, and 5y apple plants. **(b)** Relative expression levels of *MdGASA1* in apple leaves in response to exogenous 10 μM GA3 treatment. **(c)** Relative expression levels of *MdGASA1* in apple leaves in response to 50 uM sorbitol feeding. Values with different letters are significantly different according to one-way ANOVA followed by Tukey’s test (P < 0.05). Data are means ± SD (n=3).


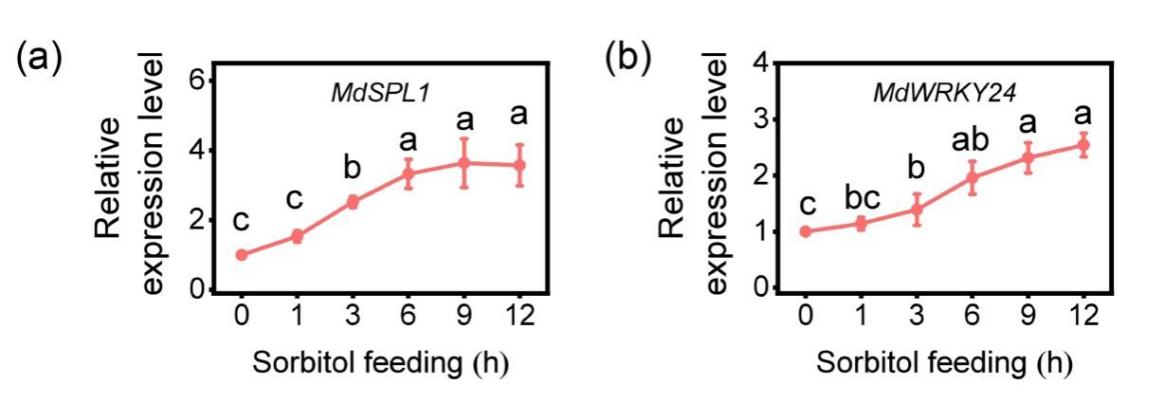


**Supplemental Figure S9** **Expression of *MdSPL1* and *MdWRKY24* in apple leaves following sorbitol treatment.** **(a)** Relative expression levels of *MdSPL1*, and **(b)** *MdWRKY24* in apple leaves in response to 50 uM sorbitol feeding. Values with different letters are significantly different according to one-way ANOVA followed by Tukey’s test (P < 0.05).

Data are means ± SD (n=3).
