# Supplementary material for: Sorbitol mediates age-dependent changes in apple plant growth strategy through gibberellin signaling
Source: Hortic Res. 2024 Jul 11;11(8):uhae192. doi: 10.1093/hr/uhae192 (PMC11322524; doi:10.1093/hr/uhae192)
Supplement: Web_Material_uhae192 [file web_material_uhae192.zip › Supplementary Table S1.pdf]

Supplementary Table S4   The primers used in this study

Annotation	Primer name	Sequence(5'-3')
Y1H analysis	MdSDH1-pro-pHIS2-F	CTTGAATTCGAGCTCGGTACC aacaagtttctccaaaaaggagagag
	MdSDH1-pro-pHIS2-R	AGCACATGCCTCGAGGTCGAC tttttttactcttctctggtttttttatt
	MdGASA1-pro-pHIS2-F	CTTGAATTCGAGCTCGGTACC TCGTTCATTGTATATTGTGCGGT
	MdGASA1-pro-pHIS2-R	AGCACATGCCTCGAGGTCGAC AGTTTGGCTTGTATGTCGTGAA
Y2H analysis	MdSPL1-BD-F	GCCATGGAGGCCGAATTC ATGGAGGGCAAGAACTTTGAAGGAAG
	MdSPL1-BD-R	CTGCAGGTCGACGGATCC TTATCTGATCTGGCAATGCTTGGAAGAAG
	MdWRKY24-AD-F	ATGGAGGCCAGTGAATTC atgaccaccaaaactctccaaaa
	MdWRKY24-AD-R	CTCGAGCTCGATGGATCC ttaccatttcttggcattaggttc
Dual-luciferase analysis	MdSDH1-pro-0800-F	gtcgacggtatcgataagctt aacaagtttctccaaaaaggagagag
	MdSDH1-pro-0800-R	cgctctagaactagtggatcc tttttttactcttctctggtttttttatt
	MdGASA1-pro-0800-F	gtcgacggtatcgataagctt TCGTTCATTGTATATTGTGCGGT
	MdGASA1-pro-0800-R	cgctctagaactagtggatcc AGTTTGGCTTGTATGTCGTGAA
	MdSPL1-62SK-F	cgctctagaactagtggatcc ATGGAGGGCAAGAACTTTGAAGGAAG
	MdSPL1-62SK-R	gataagcttgatatcgaattc TTATCTGATCTGGCAATGCTTGGAAGAAG
	MdWRKY24-62SK-F	cgctctagaactagtggatcc atgaccaccaaaactctccaaaa
	MdWRKY24-62SK-R	gataagcttgatatcgaattc ttaccatttcttggcattaggttc
Protein purification	MdSPL1-pET32a-F	ATGGCTGATATCGGATCC ATGGAGGGCAAGAACTTTGAAGGAAG
	MdSPL1-pET32a-R	GTGGTGGTGGTGGTGCTCGAGTTATCTGATCTGGCAATGCTTGGAAGAAG
	MdWRKY24-pGEX-4T-1-F	CGTGGATCCCCGGAATTC atgaccaccaaaactctccaaaa
	MdWRKY24-pGEX-4T-1-R	ACGATGCGGCCGCTCGAG ttaccatttcttggcattaggttc
	MdSPL1-pGEX-4T-1-F	CGTGGATCCCCGGAATTC ATGGAGGGCAAGAACTTTGAAGGAAG
	MdSPL1-pGEX-4T-1-R	ACGATGCGGCCGCTCGAG TTATCTGATCTGGCAATGCTTGGAAGAAG
EMSA probes	MdSDH1-(GTAC)-F	TTTTACATAGCTGTACAAGTGCAACTTG
	MdSDH1-(GTAC)-R	CAAGTTGCACTTGTACAGCTATGTAAAA
	MdSDH1-(W-box)-F	ACTTATATTGCTTGACTCTTCTCTTTAA
	MdSDH1-(W-box)-R	TTAAAGAGAAGAGTCAAGCAATATAAGT
	MdGASA1-GTAC-F	ATGATTTTTCCCCGTACGATGTACG
	MdGASA1-GTAC-R	CGTACATCGTACGGGGAAAAATCAT
	MdGASA1-W-box-F	ATGATTTTTGACCAAACGATGTACG
	MdGASA1-W-box-R	CGTACATCGTTTGGTCAAAAATCAT
qRT-PCR	MdSPL1-F	CGTCTTGTCAAGTGGAGAGGTGTG
	MdSPL1-R	GCTGGCAAAACCGCTGTTGAATC
	MdSDH1-F	TGGCTGCTTGGCTTGTTGATG
	MdSDH1-R	TCACTTCCACAAATGCCGACAG
	MdGASA1-F	TTAGATTGTGGAGTGGCATGTGAAG
	MdGASA1-R	ATAGCAAGGACAAGACTCGTAGTTG
	MdWRKY24-F	AAGTGCGGACGGGATGAATATGAAC
	MdWRKY24-R	ATGTGATGGCGATGTGGTAAGGTC
